# The Response of Soil Respiration to Temperature and Humidity in the Thermokarst Depression Zone of the Headwater Wetlands of Qinghai Lake

**DOI:** 10.3390/biology13060437

**Published:** 2024-06-14

**Authors:** Yahui Mao, Kelong Chen, Wei Ji, Yanli Yang

**Affiliations:** 1 Qinghai Province Key Laboratory of Physical Geography and Environmental Process, College of Geographical Science, Qinghai Normal University, Xining 810008, China; zealmac@163.com (Y.M.); 18909781391@163.com (Y.Y.); 2 Key Laboratory of Tibetan Plateau Land Surface Processes and Ecological Conservation (Ministry of Education), Qinghai Normal University, Xining 810008, China; 3 National Positioning Observation and Research Station of Qinghai Lake Wetland Ecosystem in Qinghai, National Forestry and Grassland Administration, Haibei 812300, China; 4 Lianyungang Academy of Agricultural Sciences, Lianyungang 222006, China

**Keywords:** Qinghai–Tibet Plateau, thermokarst, climate change, carbon cycle, soil respiration

## Abstract

**Simple Summary:**

Climate warming has caused the active layer of permafrost to thicken, leading to permafrost melting and surface collapse, forming thermokarst landforms. These changes significantly affect regional vegetation, soil properties, and water processes, thereby impacting regional carbon cycling. This study examined the relationships between soil respiration rate (Rs), soil temperature (T), and volumetric water content (VWC) in the thermokarst depression zones of Qinghai Lake’s headwater wetlands. The results showed a significant positive correlation between soil temperature and Rs, and a significant negative correlation between VWC and Rs. The inhibitory effect of VWC on Rs was stronger in thermokarst areas compared to natural conditions. Temperature had a greater influence on Rs, especially during the day, while VWC inhibited Rs more at night. The study highlights the combined impact of temperature and humidity on soil respiration, revealing that Rs in thermokarst areas is more sensitive to temperature changes at night. These findings improve our understanding of carbon cycling in wetland ecosystems and help predict wetland carbon emissions under climate change.

**Abstract:**

As the climate warms, the thickening of the active layer of permafrost has led to permafrost melting and surface collapse, forming thermokarst landforms. These changes significantly impact regional vegetation, soil physicochemical properties, and hydrological processes, thereby exacerbating regional carbon cycling. This study analyzed the relationship between soil respiration rate (Rs), soil temperature (T), and volumetric water content (VWC) in the thermokarst depression zone of the headwater wetlands of Qinghai Lake, revealing their influence on these soil parameters. Results showed a significant positive correlation between soil temperature and Rs (*p* < 0.001), and a significant negative correlation between VWC and Rs (*p* < 0.001). The inhibitory effect of VWC on Rs in the thermokarst depression zone was stronger than under natural conditions (*p* < 0.05). Single-factor models indicated that the temperature-driven model had higher explanatory power for Rs variation in both the thermokarst depression zone (R^2^ = 0.509) and under natural conditions (R^2^ = 0.414), while the humidity-driven model had lower explanatory power. Dual-factor models further improved explanatory power, slightly more so in the thermokarst depression zone. This indicates that temperature and humidity jointly drive Rs. Additionally, during the daytime, temperature had a more significant impact on Rs under natural conditions, while increased VWC inhibited Rs. At night, the positive correlation between Rs and temperature in the thermokarst depression zone increased significantly. The temperature sensitivity (Q_10_) values of Rs were 3.32 and 1.80 for the thermokarst depression zone and natural conditions, respectively, indicating higher sensitivity to temperature changes at night in the thermokarst depression zone. This study highlights the complexity of soil respiration responses to temperature and humidity in the thermokarst depression zone of Qinghai Lake’s headwater wetlands, contributing to understanding carbon cycling in wetland ecosystems and predicting wetland carbon emissions under climate change.

## 1. Introduction

Thermokarst collapses, thermokarst subsidence, and thermokarst ponds are common types of landforms in thermokarst terrain [1,2]. These landforms exert significant thermal erosion effects on their development areas and adjacent permafrost, mainly manifested by releasing large amounts of heat and accelerating the melting of nearby permafrost [3,4]. Among them, thermokarst collapses and thermokarst gullies mainly affect the shallow water and thermal conditions. The impact range of thermokarst ponds is broader, possibly leading to the formation of permafrost thaw zones beneath the ponds [5]. The emergence of thermokarst terrain exacerbates the destruction in cold permafrost regions. It severely affects alpine meadows, altering soil microenvironments, and significantly affecting CO_2_ and CH_4_ emissions from the soil [6,7]. In recent decades, the number and area of thermokarst landforms on the Qinghai–Tibet Plateau have rapidly increased [8,9]. For example, from 2008 to 2017, the total number of melting collapses in the northern foothills river basin increased by 253%, and the affected total area increased by 617%; from 2009 to 2016, the area of thermokarst landforms in the upstream of the Heihe River in the Qilian Mountains significantly increased [10]; from 1991 to 2020, the area of thermokarst ponds along the Qinghai–Tibet Highway increased from 107.14 square kilometers to 184.92 square kilometers, with the highest number of thermokarst ponds in alpine meadow vegetation types [11]. These changes have significant implications for plateau carbon cycling [12,13].

In the thermokarst depression zone of the headwater wetlands of Qinghai Lake, the response of soil respiration to temperature and humidity has become a highly scrutinized research topic. With the exacerbation of global climate change, wetland ecosystems, as crucial factors in maintaining the Earth’s ecological balance, are gaining increasing importance [14,15]. As an important wetland in China, the headwater wetlands of Qinghai Lake hold significant ecological value in terms of their response to climate change and the maintenance of their ecological functions [16,17]. Global warming is significantly altering the temperature and humidity environment of wetland ecosystems, profoundly affecting the biological processes in wetland soils, particularly the process of soil respiration [18,19,20]. Soil respiration, as a crucial component of carbon cycling in wetland ecosystems, directly influences the dynamic changes of soil carbon pools and greenhouse gas emissions [21,22]. Therefore, in-depth research into the response mechanisms of soil respiration to changes in temperature and humidity is of great significance for understanding the carbon dynamics of wetland ecosystems and their response to climate change. This paper aims to systematically investigate the response of soil respiration to temperature and humidity in the thermokarst depression zone of the headwater wetlands of Qinghai Lake. It also seeks to explore soil respiration patterns under various temperature and humidity conditions and clarify the correlation mechanisms between soil respiration and temperature and humidity. Through these studies, theoretical foundations and scientific references are provided for research on the carbon cycling of wetland ecosystems. This not only contributes to the protection and restoration of wetland ecosystems but also offers theoretical and technical support for addressing climate change.

## 2. Materials and Methods

### 2.1. Experimental Site Description

**Figure 1:**
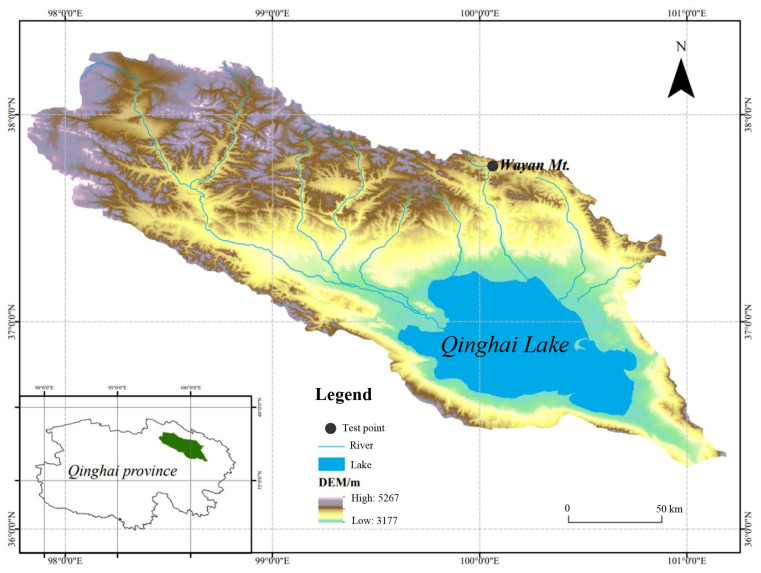
Experimental site locations in the Qinghai Lake basin and on the northern shore of Qinghai Lake.

The experimental site is located at Wayan Mountain (37.74° N, 100.09° E) in Yike Wulan Township, Gangcha County, Haibei Tibetan Autonomous Prefecture, on the northern shore of Qinghai Lake (Figure 1). It belongs to the river source wetland and is situated near Qinghai Lake, within the Datong Mountain Range of the Qilian Mountains. The test point is at an altitude of 3700–3850 m above sea level, and the soil type belongs to the semi-hydrous soil order, characterized as meadow soil.

### 2.2. Soil Respiration Measurement and Data Collection

Soil respiration measurement: Using the Soil CO_2_ Measurement System Li-8100 (manufactured by LI-COR in Lincoln, NE, USA), short-term observations of soil respiration were conducted in the thermal subsidence zone of the Qinghai Lake River Source Wetland on 21 July, 16 August, and 10 September 2017, under clear weather conditions [23]. Observations were carried out throughout a 24 h daily cycle, with measurements obtained every 2 h (every 3 h from 0:00 to 06:00). Prior to the measurements, aboveground vegetation within the soil respiration collar was removed the day before to minimize the soil disturbance. Soil temperature at 5 cm depth and volumetric water content were measured using the built-in temperature and humidity sensors of the Li-8100 (manufactured by LI-COR in Lincoln, NE, USA).

Meteorological factors determination: Surface soil temperature was measured by the 8100-203 soil temperature sensor connected to the measurement chamber. The volumetric water content (VWC) of surface soil, which is the ratio of the volume of water contained in a unit volume of soil to the total volume of that unit of soil, was measured by the EC-5 soil moisture sensors (manufactured by Decagon Devices in Pullman, Washington, USA). connected to the monitoring station. The volumetric water content represents the unfrozen water content [24]. Air temperature (Tair), precipitation, surface temperature (Tsoil), atmospheric relative humidity (RH), and other meteorological data were measured by the CR1000 research-grade automatic weather station(manufactured by Dynamax in Houston, Texas, USA); these data were automatically recorded every half hour. Electrical conductivity of soluble salts (EC) was determined by the EM50 (manufactured by Decagon Devices in Pullman, Washington, USA, sensor model 5TE), with measurements obtained every half hour, and the experiment was conducted with 6 repetitions.

### 2.3. Determination of Aboveground and Belowground Biomass and Soil Organic Matter

During the peak growth period in August, sampling was conducted randomly in both the thermal subsidence area and the natural state using quadrats measuring 20 × 20 cm. Aboveground vegetation was harvested, including litter, and placed into parchment envelopes, which were brought back to the laboratory. After being dried at 105 °C in a constant temperature convection oven for 15 min to halt biological activity, the samples were dried at 65 °C for 24 h and weighed. The average weight of 100 parchment envelopes was calculated to obtain the aboveground biomass indicator after unit conversion [25].

When collecting aboveground biomass samples, a root auger with a diameter of 10.5 cm was used to extract soil at depths of 0–10 cm, 10–20 cm, and 20–30 cm. After soil samples were obtained, the holes were refilled with nearby soil from the same plot. The extracted soil samples were placed on a sieve with a pore size of 0.28 mm to separate the roots from the soil. The root systems were washed with water, and live roots were identified based on color and flexibility. Live roots were then placed in a constant temperature convection oven at 75 °C until a constant weight was achieved. After drying, the underground biomass was calculated based on measurements obtained using a 1/1000 balance [26].

Soil organic matter determination involved sampling and processing soil from eight designated points in both the thermal subsidence area and the natural state. Soil samples were collected at depths of 0–10 cm, 10–20 cm, and 20–30 cm, and after sampling, the holes were refilled with nearby soil from the same plot. The soil samples were then naturally air-dried in a shaded and ventilated area of the laboratory and subsequently ground and passed through a sieve with a pore size of 0.149 mm. Subsequently, silk and a glass rod were used to rub the soil samples, and a glass rod with static electricity was used to adsorb tiny plant residues from the soil samples. An amount of 0.1 g of soil sample (accurate to 0.0001 g) was weighed on a 1/1000 analytical balance, and the determination of organic matter was conducted using the potassium dichromate–oil bath method [27]. An average of six repetitions was taken for each result.

### 2.4. Statistical Analysis

The variance analysis and correlation analysis of this study were conducted using SPSS 26.0. One-way analysis of variance (ANOVA) was employed to compare the mean differences in soil respiration rates under different treatments. For the various treatments, thermal factors were subjected to one-way ANOVA, and multiple comparisons were performed using the LSD method for minimum significant difference. Spearman’s rank correlation coefficient and Pearson correlation analysis were utilized to analyze the correlation coefficients between soil respiration rate (Rs) and influencing factors such as temperature and volumetric water content. A stepwise linear regression model was used to explore the contribution of relevant thermal factors to the variation in soil respiration rate (Rs). Regression analysis was performed on soil respiration (Rs) and its influencing factors using empirical models. The fitting function with higher goodness of fit was selected as the optimal regression equation. Regression analysis and plotting were carried out using OriginPro 2018.

### 2.5. Data Processing

#### 2.5.1. Commonly Used Functional Equations for the Relationship between Soil Respiration Rate and Soil Temperature (Modified)

Exponential model (vant’ Hoff): $R_{s}=ae^{bT}$ [28].

“a” is the soil respiration rate at a temperature of 0, “b” is the temperature response coefficient, “T” is the temperature, and “Rs” is the soil respiration rate at temperature T (measured in μmol·m^−2^·s^−1^).

$$Q_{10}=e^{10b}$$

Q_10_ is the temperature sensitivity coefficient of soil respiration, representing the factor by which soil respiration increases for every 10 °C rise in soil temperature.

Low-temperature power function (Kucera and Kirkham): $R_{s}=a{\left(T+10\right)}^{b}$ [29].

“a” and “b” are fitting parameters, “T” is the temperature, and “Rs” is the soil respiration rate at temperature T (measured in μmol·m^−2^·s^−1^).

#### 2.5.2. Common Functional Models for the Relationship between Soil Respiration Rate and Soil Volumetric Water Content

The common functional models for the relationship between soil respiration rate and soil volumetric water content are as follows:

$$R_{s}=a+bW\;R_{s}=a+bW+cW^{2}$$

$R_{s}=\exp\left({-e}^{\left(p-qW\right)}\right)$ [30].

“a”, “b”, “c”, “p”, and “q” are fitting parameters, “W” is the soil volumetric water content, and “Rs” is the soil respiration rate (measured in μmol·m^−2^·s^−1^).

## 3. Results

### 3.1. Dynamic Characteristics of Soil Respiration in Thermokarst Areas

**Figure 2:**
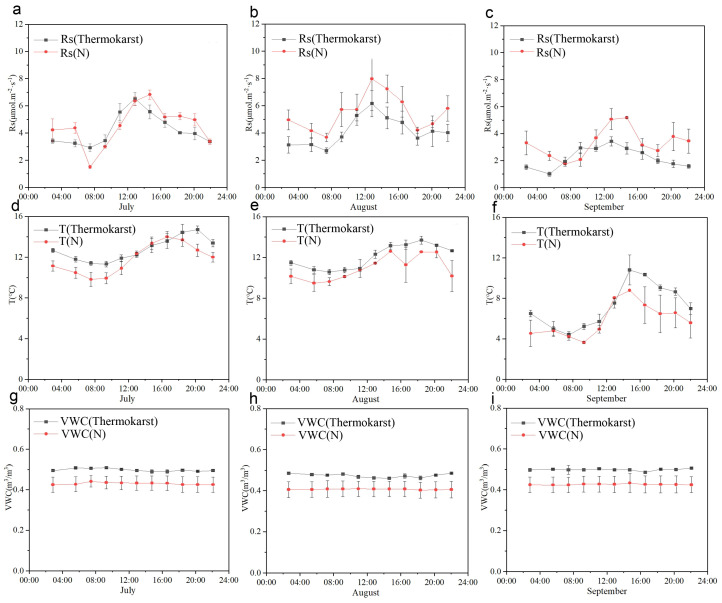
Daily dynamic variation characteristics of soil respiration rate in thermokarst and natural states; (**a**–**c**) depict the daily variations in soil respiration rate for July, August, and September, respectively, in both thermokarst areas and under natural conditions; (**d**–**f**) illustrate the daily variations in soil temperature for July, August, and September, respectively, in both thermokarst areas and under natural conditions; (**g**–**i**) display the daily variations in soil volumetric water content for July, August, and September, respectively, in both thermokarst areas and under natural conditions; “N” represents soil under natural conditions, and “Thermokarst” represents soil in thermokarst areas.

Based on the data from Figure 2, during the observation period in July (Figure 2a,d,g), the highest soil respiration rate (Rs, Thermokarst) in the thermokarst area was 6.60 μmol·m^−2^·s^−1^, occurring at 14:00, with a daily average of 4.37 μmol·m^−2^·s^−1^. The average soil temperature (T, Thermokarst) was 13.11 °C, and the average soil volumetric water content (VWC, Thermokarst) was 0.497 m^3^/m^3^. In contrast, under natural conditions, the highest soil respiration rate (Rs, N) was 6.91 μmol·m^−2^·s^−1^, occurring at 16:00, with a daily average of 4.66 μmol·m^−2^·s^−1^. The average soil temperature (T, N) was 13.11 °C, and the average soil volumetric water content (VWC, N) was 0.497 m^3^/m^3^.

During the observation period in August (Figure 2b,e,h), the highest soil respiration rate in the thermokarst area was 6.24 μmol·m^−2^·s^−1^, occurring at 14:00, with a daily average of 4.205 μmol·m^−2^·s^−1^. The average soil temperature was 12.46 °C, and the average soil volumetric water content was 0.477 m^3^/m^3^. Under natural conditions, the highest soil respiration rate was 8.1 μmol·m^−2^·s^−1^, occurring at 14:00, with a daily average of 5.56 μmol·m^−2^·s^−1^. The average soil temperature was 11.31 °C, and the average soil volumetric water content was 0.41 m^3^/m^3^.

During the observation period in September (Figure 2c,f,i), the highest soil respiration rate in the thermokarst area was 3.46 μmol·m^−2^·s^−1^, occurring at 14:00, with a daily average of 2.28 μmol·m^−2^·s^−1^. The average soil temperature was 7.46 °C, and the average soil volumetric water content was 0.499 m^3^/m^3^. Under natural conditions, the highest soil respiration rate was 5.21 μmol·m^−2^·s^−1^, occurring at 16:00, with a daily average of 3.37 μmol·m^−2^·s^−1^. The average soil temperature was 6.02 °C, and the average soil volumetric water content was 0.428 m^3^/m^3^.

### 3.2. Soil Physicochemical Properties and Biomass in Thermokarst Areas

According to Table 1, many different contents were analyzed during the observation period. The soil respiration rate under natural conditions was 4.52 μmol·m^−2^·s^−1^, whereas in the thermokarst area, it was 3.59 μmol·m^−2^·s^−1^. The soil respiration rate in the thermokarst area was significantly lower than that under natural conditions (*p* < 0.05).

The soil volumetric water content under natural conditions was 0.42 m^3^/m^3^, whereas in the thermokarst area, it was 0.49 m^3^/m^3^. The soil volumetric water content in the thermokarst area was significantly higher than that under natural conditions (*p* < 0.05). The soil temperature under natural conditions was 9.85 °C, while in the thermokarst area, it was 11.03 °C. Although the soil temperature in the thermokarst area was higher than that under natural conditions, the difference was not significant (*p* > 0.05). In the aboveground biomass, the results showed that the aboveground biomass under natural conditions was 257.72 g/m^2^, whereas in the thermokarst area, it was 186.34 g/m^2^. The aboveground biomass in the thermokarst area was significantly lower than that under natural conditions (*p* < 0.05).

**Figure 3:**
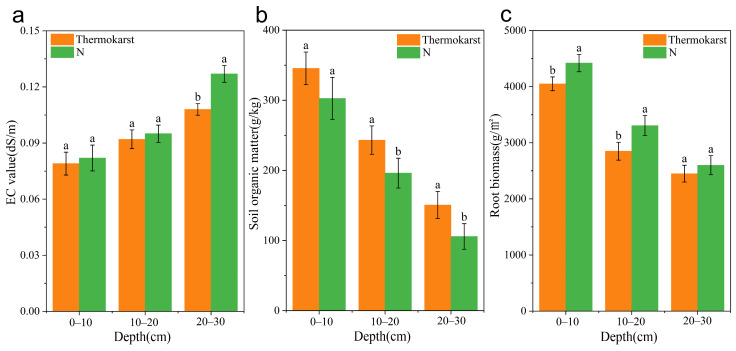
Soil physicochemical properties and biomass under thermokarst subsidence and natural conditions; “N” represents soil under natural conditions, and “Thermokarst” represents soil in thermokarst areas. (**a**) soil electrical conductivity (EC) values at different depths under thermokarst subsidence and natural conditions; (**b**) soil organic matter at different depths under thermokarst subsidence and natural conditions; (**c**) belowground root biomass at different depths under thermokarst subsidence and natural conditions; The same lowercase letters indicate that the differences in soil ecological indicators between thermokarst and natural conditions during the same period are not significant (*p* > 0.05). Different lowercase letters indicate that the differences in soil ecological indicators between thermokarst and natural conditions during the same period are significant (*p* < 0.05).

At the same times, soil electrical conductivity (EC) was also analyzed, in the 0–10 cm, 10–20 cm, and 20–30 cm soil layers, the EC values under natural conditions were 0.082 dS/m, 0.095 dS/m, and 0.127 dS/m, respectively, while in the thermokarst area, they were 0.079 dS/m, 0.092 dS/m, and 0.108 dS/m, respectively. The EC values increased with depth. In the 20–30 cm soil layer, the EC value in the thermokarst area was significantly lower than that under natural conditions (*p* < 0.05) (Figure 3a). In the 0–10 cm, 10–20 cm, and 20–30 cm soil layers, the soil organic matter content under natural conditions was 302.63 g/kg, 196.17 g/kg, and 105.86 g/kg, respectively, while in the thermokarst area, it was 345.52 g/kg, 243.25 g/kg, and 150.71 g/kg, respectively. The soil organic matter content decreased with depth. In the 10–20 cm and 20–30 cm soil layers, the organic matter content in the thermokarst area was significantly higher than that under natural conditions (*p* < 0.05) (Figure 3b).

In the soil root biomass, the result showed that in the 0–10 cm, 10–20 cm, and 20–30 cm soil layers, the root biomass under natural conditions was 4422 g/m^2^, 3306 g/m^2^, and 2601 g/m^2^, respectively, while in the thermokarst area, it was 4053 g/m^2^, 2857 g/m^2^, and 2457 g/m^2^, respectively. The root biomass decreased with depth. In the 0–10 cm and 10–20 cm soil layers, the root biomass in the thermokarst area was significantly lower than that under natural conditions (*p* < 0.05). In the 20–30 cm soil layer, the root biomass in the thermokarst area was lower than that under natural conditions, but the difference was not significant (*p* > 0.05) (Figure 3c).

### 3.3. Fitting of Single-Factor Driven Models of Rs with Temperature and Humidity in the Thermokarst Subsidence Zone

**Figure 4:**
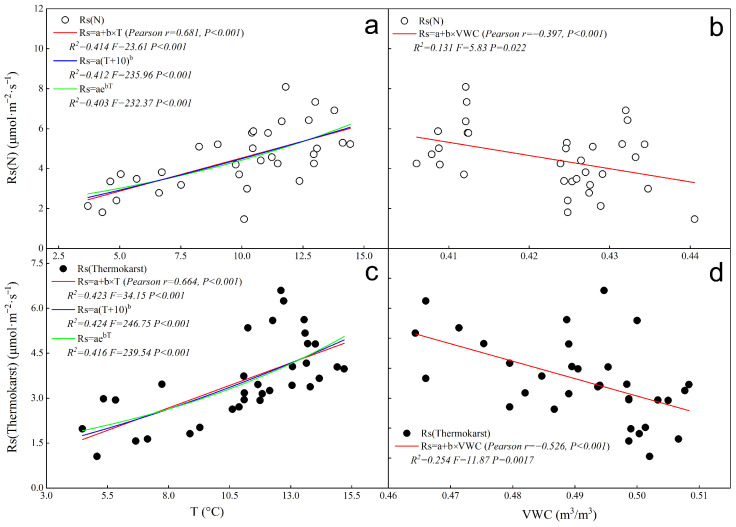
Fitting of single-factor driven models of soil respiration (Rs) with soil temperature and humidity in the thermokarst subsidence zone and natural conditions; “N” represents soil under natural conditions, and “Thermokarst” represents soil in thermokarst areas. (**a**) single-factor driven model fitting of soil respiration (Rs) with soil temperature in natural conditions; (**b**) single-factor driven model fitting of soil respiration (Rs) with soil humidity in natural conditions; (**c**) single-factor driven model fitting of soil respiration (Rs) with soil temperature in the thermokarst subsidence zone; (**d**) single-factor driven model fitting of soil respiration (Rs) with soil humidity in the thermokarst subsidence zone.

According to the data shown in Figure 4, during the observation period, the correlation coefficient (Pearson) between soil respiration rate (Rs) and soil temperature (T) in the thermokarst subsidence zone was r = 0.664, *p* < 0.001 (Figure 4c), and in the natural state was r = 0.681, *p* < 0.001 (Figure 4a). The correlation coefficient between soil respiration rate and volumetric water content (VWC) was r = −0.526, *p* < 0.001 in the thermokarst subsidence zone (Figure 4d), and r = −0.397, *p* < 0.05 in the natural state (Figure 4b). Both in the thermokarst subsidence zone and in the natural state, soil respiration rate (Rs) showed a significant positive correlation with soil temperature and a significant negative correlation with volumetric water content. However, the inhibitory effect of volumetric water content on Rs in the thermokarst subsidence zone was significantly stronger than in the natural state (*p* < 0.05).

When analyzing the fitting effect of single-factor driven models of Rs with soil temperature, it was found that there was little difference in the fitting effect of several single-factor temperature-driven models. Among them, the simple linear regression model showed a higher degree of fitting for the temperature and Rs relationship, with an R^2^ of 0.509 (*p* < 0.001) in the thermokarst subsidence zone and R^2^ of 0.414 (*p* < 0.001) in the natural state.

Regarding the fitting relationship between Rs and soil volumetric water content, the simple linear regression model explained 25.4% of the variation in Rs in the thermokarst subsidence zone and 13.1% in the natural state.

### 3.4. Fitting of Dual-Factor Driven Models of Rs with Temperature and Humidity in the Thermokarst Subsidence Zone

**Figure 5:**
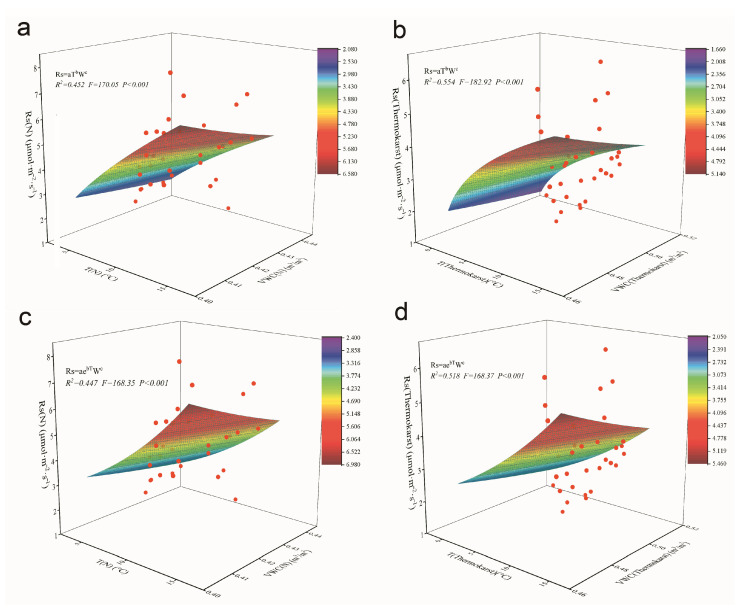
Fitting of dual-factor models of soil respiration with temperature and humidity in the thermokarst subsidence zone and natural conditions; “N” represents soil under natural conditions, and “Thermokarst” represents soil in thermokarst areas. (**a**) fitting of dual-factor driven model of soil respiration (Rs) with soil temperature and humidity in natural conditions; (**b**) fitting of dual-factor driven model of soil respiration (Rs) with soil temperature and humidity in the thermokarst subsidence zone; (**c**) fitting of dual-factor driven model of soil respiration (Rs) with soil temperature and humidity in natural conditions; (**d**) fitting of dual-factor driven model of soil respiration (Rs) with soil temperature and humidity in the thermokarst subsidence zone.

According to the results from Figure 5, the two dual-factor models incorporating temperature and humidity demonstrate good fitting in explaining variations in soil respiration (Rs) both in the thermokarst subsidence zone and natural conditions. The first model explains 45.2% of the variation in soil respiration in natural conditions (Figure 5a) and 55.4% in the thermokarst subsidence zone (Figure 5b). The second model explains 44.7% of the variation in soil respiration in natural conditions (Figure 5c) and 51.8% in the thermokarst subsidence zone (Figure 5d). Both models show consistent explanatory power across different environments, with slightly better performance in the thermokarst subsidence zone. This indicates that temperature and humidity are crucial factors influencing soil respiration, and the effectiveness of the models has been validated across different environmental conditions. The high fitting of the models suggests that the selected dual factors of temperature and humidity have strong explanatory power in predicting soil respiration.

### 3.5. The Impact of Diurnal Variations in Thermokarst Regions on Soil Ecological Indicators

According to Table 2, concerning soil temperature, both during the day and at night, the soil temperature in natural conditions is lower than in the thermokarst subsidence zone, but the difference is not significant (*p* > 0.05). Regarding soil volumetric water content, both during the day and at night, the soil volumetric water content in natural conditions is lower than in the thermokarst subsidence zone, and the soil volumetric water content in the thermokarst subsidence zone is significantly higher than in natural conditions (*p* < 0.05). In terms of soil respiration rate both during the day and at night, the soil respiration rate in natural conditions is higher than in the thermokarst subsidence zone. The difference during the day is not significant (*p* > 0.05), while the difference at night is significant (*p* < 0.05). Regardless of whether it is in natural conditions or the thermokarst subsidence zone, the soil respiration rate is higher during the day than at night. Specifically, the average daytime and nighttime soil respiration rates in the thermokarst subsidence zone are 4.09 μmol·m^−2^·s^−1^ and 2.82 μmol·m^−2^·s^−1^, respectively, with a daytime increase of 45.04% over nighttime. In natural conditions, the average daytime and nighttime soil respiration rates are 4.77 μmol·m^−2^·s^−1^ and 4.15 μmol·m^−2^·s^−1^, respectively, with a daytime increase of 14.94% over nighttime.

### 3.6. Fitting Single-Factor Models for Soil Respiration in Thermokarst and Natural Zones

**Figure 6:**
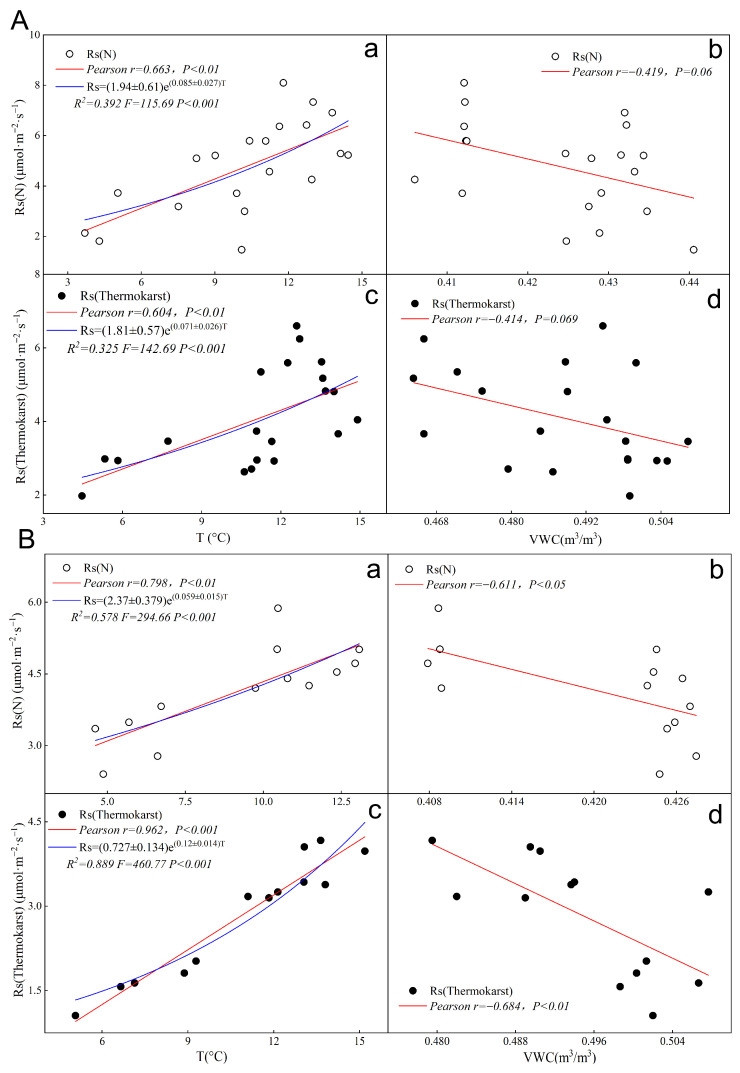
Fitting of single-factor driven models of soil respiration rate with temperature and humidity during daytime and nighttime in the thermokarst subsidence zone and natural conditions; “N” represents soil under natural conditions, and “Thermokarst” represents soil in thermokarst areas. (**Aa**) fitting of single-factor driven model of soil respiration rate with temperature during daytime in natural conditions; (**Ab**) fitting of single-factor driven model of soil respiration rate with humidity during daytime in natural conditions; (**Ac**) fitting of single-factor driven model of soil respiration rate with temperature during daytime in the thermokarst subsidence zone; (**Ad**) fitting of single-factor driven model of soil respiration rate with humidity during daytime in the thermokarst subsidence zone; (**Ba**) fitting of single-factor driven model of soil respiration rate with temperature during nighttime in natural conditions; (**Bb**) fitting of single-factor driven model of soil respiration rate with humidity during nighttime in natural conditions; (**Bc**) fitting of single-factor driven model of soil respiration rate with temperature during nighttime in the thermokarst subsidence zone; (**Bd**) fitting of single-factor driven model of soil respiration rate with humidity during nighttime in the thermokarst subsidence zone.

During the day, the Pearson correlation coefficient between soil respiration rate (Rs) and soil temperature (T) in the thermokarst subsidence zone is r = 0.604, *p* < 0.001 (Figure 6Ac), while in natural conditions, it is r = 0.663, *p* < 0.001 (Figure 6Aa). The correlation coefficient between soil respiration rate and volumetric water content (VWC) is r = −0.414, *p* < 0.001 in the thermokarst subsidence zone (Figure 6Ad), and r = −0.419, *p* < 0.01 in natural conditions (Figure 6Ab). The soil respiration temperature sensitivity (Q_10_) in the thermokarst subsidence zone and natural conditions are 2.03 and 2.34, respectively (Table 2).

During the night, the Pearson correlation coefficient between soil respiration rate and soil temperature in the thermokarst subsidence zone is r = 0.962, *p* < 0.001 (Figure 6Bc), while in natural conditions, it is r = 0.798, *p* < 0.001 (Figure 6Ba). The correlation coefficient between soil respiration rate and volumetric water content is r = −0.684, P < 0.01 in the thermokarst subsidence zone (Figure 6Bd), and r = −0.611, *p* < 0.05 in natural conditions (Figure 6Bb). The soil respiration temperature sensitivity (Q_10_) in the thermokarst subsidence zone and natural conditions are 3.32 and 1.80, respectively (Table 2).

From the perspective of day and night, both the positive and negative correlations between soil temperature (T) and volumetric water content (VWC) with soil respiration rate (Rs) are strengthened during the night in the thermokarst subsidence zone and natural conditions. Additionally, the soil respiration temperature sensitivity (Q_10_) in the thermokarst subsidence zone is higher during the night than in natural conditions, indicating that the response of soil respiration rate to temperature is significantly higher during the night in the thermokarst subsidence zone.

## 4. Discussion

Climate warming has led to the thickening of the active layer of permafrost, resulting in permafrost thawing, surface collapse, and the gradual accumulation of water. This has given rise to thermokarst landforms. This phenomenon has had a drastic impact on regional vegetation, soil physicochemical properties, and hydrological processes, exacerbating regional carbon cycling [12,31,32]. Remote sensing interpretation by Zhongwen revealed a significant increase in the area of thermokarst landforms in the Heihe River Basin of the Qilian Mountains. Temperature variations and extreme precipitation events are key factors in the development of thermokarst [10]. There are significant differences in carbon emissions processes among different thermokarst landforms [33]. In the study of thermokarst lake areas along the G109 in the central Tibetan Plateau by Jia Lin, the highest greenhouse gas emissions during the growing season occurred in August (27.61 mmol·m^−2^·d^−1^), while the lowest occurred in September (10.47 mmol·m^−2^·d^−1^). Moreover, carbon emissions from alpine marshy meadows were higher than those from alpine meadows, alpine deserts, and arid grasslands [34]. Mumei found in the thermokarst collapse area of the Qilian Mountains that ecosystem respiration increased by 8.5% in the early stage of thermokarst collapse (<5 years) and decreased by 25% and 40% in the middle stage (5–8 years, 23–51 years), and late stage (>51 years). This suggests that with the passage of time, the carbon absorption capacity of the ecosystem weakens, and it may even transform into a carbon source [35]. In the study of freeze–thaw erosion areas in Wayan Mountain by Ding Junxia, the soil respiration rate was lower in the freeze–thaw erosion area compared to the natural state; the rates were 1.56 µmol·m^−2^·s^−1^ and 2.95 µmol·m^−2^·s^−1^, respectively. Additionally, the soil respiration rate was positively correlated with temperature and negatively correlated with volumetric water content [36]. The results of this study are consistent with this conclusion.

Due to the significantly smaller aboveground and belowground biomass in the thermokarst subsidence zone compared to the natural state, this is also the main reason why its soil respiration rate is significantly lower than that of the natural state. Additionally, the volumetric water content in the thermokarst subsidence zone is significantly higher than in the natural state (*p* < 0.01), leading to a more pronounced inhibitory effect on soil respiration. The ecological stability of the thermokarst subsidence zone is lower [37], making it more susceptible to disturbances in temperature and moisture, resulting in its higher temperature sensitivity compared to the natural state. Furthermore, the thermokarst subsidence zone experiences higher temperatures and volumetric water content. This leads to more heat release during the freezing period and more heat absorption during the thawing period, compared to the natural state. During precipitation events, the subsidence zone accumulates a large amount of water. This exacerbates erosion of nearby vegetation, further expanding the thermokarst subsidence zone. It accelerates the release of carbon stored in the permafrost and causes lateral erosion in surrounding permafrost areas, leading to accelerated degradation of permafrost [38].

By analyzing the relationship between soil respiration rate (Rs), soil temperature (T), and volumetric water content (VWC) in thermokarst areas, we found a significant positive correlation between soil temperature and Rs (*p* < 0.001). An increase in soil temperature significantly enhances Rs, both in thermokarst areas and under natural conditions. This finding is consistent with existing research, supporting the theory that temperature is the main driver of soil respiration [39]. On the other hand, there is a significant negative correlation between Rs and volumetric water content (*p* < 0.001). However, the inhibitory effect of VWC on Rs is significantly stronger in thermokarst areas than under natural conditions (*p* < 0.05). This difference may be related to the dynamic changes in moisture in thermokarst areas and their impact on soil microbial activity and organic matter decomposition [40]. Excessive water content can lead to soil anoxia, thereby inhibiting microbial activity and reducing Rs [41].

The analysis results of the single-factor model support the view that temperature is the primary driving factor, while also emphasizing the significant role of humidity under specific conditions. The dual-factor model introduces both temperature and humidity variables, which better explain the variations in soil respiration (Rs). The explanatory power of the model remains consistent across different environments, with slightly higher performance in the thermokarst subsidence zone compared to the natural state. This indicates that temperature and humidity jointly play significant roles as driving factors for Rs. The dual-factor model provides more accurate predictions of soil respiration changes, which is crucial for understanding the soil carbon cycling process under different environmental conditions [42].

This study examined the diurnal variations of soil temperature, volumetric water content, and soil respiration rates in the thermokarst subsidence zone compared to natural conditions, revealing the impacts of the thermokarst subsidence zone on these soil parameters. The research found that regardless of day or night, the soil temperature in the thermokarst subsidence zone was higher than in the natural state, although the difference was not significant (*p* > 0.05). This could be attributed to the soil structure and thermal conductivity properties of the thermokarst subsidence zone leading to temperature increases that did not reach a significant level. Regarding soil volumetric water content, both during the day and at night, the soil in the thermokarst subsidence zone exhibited significantly higher volumetric water content compared to the natural state (*p* < 0.05). This could be due to the soil structure of the thermokarst subsidence zone being more conducive to moisture retention, resulting in higher water content. This high moisture environment may promote microbial activity but could also inhibit certain aerobic respiration processes. In terms of soil respiration rates (Rs), regardless of day or night, the soil respiration rates in the natural state were higher than in the thermokarst subsidence zone, with the impact of the thermokarst subsidence zone on soil respiration rates being more significant at night.

The study utilized a single-factor driving model to examine the correlation between soil respiration rate and temperature (T) and volumetric water content (VWC). Results indicated a significant positive correlation between soil respiration rate and temperature, and a significant negative correlation with volumetric water content, irrespective of whether in the thermokarst subsidence zone or in the natural state. During the daytime, temperature exerted a more pronounced effect on soil respiration rate in the natural state, while increased volumetric water content inhibited soil respiration rate. Conversely, during the nighttime, the positive correlation between soil respiration rate and temperature in the thermokarst subsidence zone intensified, with a more pronounced negative correlation with volumetric water content (VWC). Overall, the thermokarst subsidence zone notably influences soil temperature, volumetric water content, and soil respiration rate, particularly at night. These variations could impact carbon cycling and climate feedback mechanisms by altering soil microbial activity and respiration processes. Moreover, prolonged water accumulation in the thermokarst subsidence zone elevates volumetric water content compared to the natural state, creating an anaerobic environment that reduces aerobic microbial and plant root respiration, resulting in carbon emissions primarily from microbial heterotrophic respiration [43,44,45].

In summary, this study has revealed the complexity of soil respiration responses to temperature and humidity in the thermokarst subsidence zone of the Qinghai Lake River Source Wetland. These findings not only contribute to understanding the processes of carbon cycling in wetland ecosystems but also provide scientific evidence for predicting the dynamics of carbon emissions from wetlands under the background of climate change. Future research should further explore the mechanisms of temperature and humidity effects on soil respiration in different types of wetlands and climatic conditions to improve the accuracy of ecological models. Additionally, considering the long-term impact of thermokarst subsidence on wetland ecosystems, larger-scale and longer-term observational and simulation studies are needed to better understand and address the changes in wetland ecosystems under the background of global climate change.

## 5. Conclusions

This study has highlighted the intricate dynamics of soil respiration (Rs) in the thermokarst depression zones of the headwater wetlands of Qinghai Lake, focusing on the interactions between soil temperature (T), volumetric water content (VWC), and Rs. Key findings include the following:

(1) Temperature and Rs correlation: There is a significant positive correlation between soil temperature and Rs, with temperature demonstrating a higher explanatory power for Rs variations compared to volumetric water content.
(2) Water content and Rs correlation: Volumetric water content shows a significant negative correlation with Rs. Notably, the inhibitory effect of water content on Rs is more pronounced in thermokarst depression zones than under natural conditions.
(3) Driving factors for Rs: Single-factor models indicated that temperature-driven models have greater explanatory power for Rs variations than humidity-driven models. However, dual-factor models, which incorporate both temperature and humidity, provide an even more accurate explanation of Rs dynamics, particularly in thermokarst depression zones.
(4) Day–night variations: During the day, temperature predominantly influences Rs under natural conditions, while increased water content inhibits Rs. At night, the correlation between temperature and Rs in thermokarst depression zones strengthens significantly, with a pronounced negative impact from increased water content.
(5) Temperature sensitivity (Q_10_): The Q_10_ values for Rs in thermokarst depression zones (3.32) are higher than under natural conditions (1.80), indicating a greater sensitivity of Rs to temperature changes at night in these zones.

Overall, this study emphasizes the complexity of soil respiration responses to climatic factors in permafrost regions. It deepens our understanding of the carbon cycling processes in wetland ecosystems and provides a scientific basis for predicting carbon emission dynamics under climate change. These insights are crucial for developing effective strategies to mitigate the impacts of climate warming on these sensitive ecosystems. Furthermore, the findings significantly enrich the international scientific community’s knowledge of global carbon cycles and offer valuable information for the local government of Qinghai Province to formulate and implement policies and measures addressing the environmental challenges specific to their region.

## Figures and Tables

**Table 1:** Soil ecological indicators under thermokarst subsidence and natural conditions.

Ecological Indicators	T (°C)	VWC (m^3^/m^3^)	RS (μmol·m^−2^·s^−1^)	Above-Ground Biomass (g/m^2^)
N	9.85 ± 1.36 a	0.42 ± 0.04 b	4.52 ± 0.36 a	257.72 ± 31.23 a
Thermokarst	11.03 ± 1.69 a	0.49 ± 0.03 a	3.59 ± 0.49 b	186.34 ± 18.31 b

Note: “N” represents soil under natural conditions, and “Thermokarst” represents soil in thermokarst areas. The same lowercase letters indicate that the differences in soil ecological indicators between thermokarst and natural conditions during the same period are not significant (*p* > 0.05). Different lowercase letters indicate that the differences in soil ecological indicators between thermokarst and natural conditions during the same period are significant (*p* < 0.05).

**Table 2:** The impact of diurnal variations in thermokarst regions on soil ecological indicators.

Ecological Indicators	T (°C)		VWC (m^3^/m^3^)		RS (μmol·m^−2·^s^−1^)		Q_10_	
	Day	Night	Day	Night	Day	Night	Day	Night
N	10.05 ± 2.73a	9.01 ± 2.35a	0.416 ± 0.021b	0.413 ± 0.011b	4.77 ± 0.41a	4.15 ± 0.31a	2.34 ± 0.36a	1.81 ± 0.25b
Thermokarst	11.11 ± 2.81a	10.52 ± 2.62a	0.476 ± 0.027a	0.473 ± 0.022a	4.09 ± 0.37a	2.82 ± 0.22b	2.03 ± 0.27a	3.32 ± 0.38a

Note: “N” represents soil under natural conditions, and “Thermokarst” represents soil in thermokarst areas. The same lowercase letters indicate that the differences in soil ecological indicators between thermokarst and natural conditions during the same period are not significant (*p* > 0.05). Different lowercase letters indicate that the differences in soil ecological indicators between thermokarst and natural conditions during the same period are significant (*p* < 0.05).

## Data Availability

All data generated or analyzed during this study are included in this published article.
